# Tumor Necrosis Factor-α-Induced Protein 8-Like 2 Negatively Regulates Innate Immunity Against RNA Virus by Targeting RIG-I in Macrophages

**DOI:** 10.3389/fimmu.2021.642715

**Published:** 2021-03-19

**Authors:** Ziqi Zou, Mengyao Li, Yunlian Zhou, Jiaying Li, Ting Pan, Lihua Lai, Qingqing Wang, Lining Zhang, Qun Wang, Yinjing Song, Yuanyuan Zhang

**Affiliations:** ^1^Institute of Immunology, Zhejiang University School of Medicine, Hangzhou, China; ^2^The Children's Hospital, Zhejiang University School of Medicine, Hangzhou, China; ^3^Department of Immunology, School of Basic Medical Science, Shandong University, Jinan, China; ^4^Sir Run Run Shaw Hospital, Zhejiang University School of Medicine, Hangzhou, China

**Keywords:** TIPE2, type I interferon, RIG-I, VSV, macrophage

## Abstract

A systematic and flexible immunoregulatory network is required to ensure the proper outcome of antiviral immune signaling and maintain homeostasis during viral infection. Tumor necrosis factor-α-induced protein 8-like 2 (TIPE2), a novel immunoregulatory protein, has been extensively studied in inflammatory response, apoptosis, and cancer. However, the function of TIPE2 in antiviral innate immunity is poorly clarified. In this study, we reported that the expression of TIPE2 declined at the early period and then climbed up in macrophages under RNA virus stimulation. Knockout of TIPE2 in the macrophages enhanced the antiviral capacity and facilitated type I interferon (IFN) signaling after RNA viral infection both *in vitro* and *in vivo*. Consistently, overexpression of TIPE2 inhibited the production of type I IFNs and pro-inflammatory cytokines, and thus promoted the viral infection. Moreover, TIPE2 restrained the activation of TBK1 and IRF3 in the retinoic acid inducible gene-I (RIG-I)-like receptors (RLR) signaling pathway by directly interacting with retinoic acid inducible gene-I (RIG-I). Taken together, our results suggested that TIPE2 suppresses the type I IFN response induced by RNA virus by targeting RIG-I and blocking the activation of downstream signaling. These findings will provide new insights to reveal the immunological function of TIPE2 and may help to develop new strategies for the clinical treatment of RNA viral infections.

## Introduction

Innate immunity constitutes the front line of host immune defense against invading exogenous pathogens by detecting diverse pathogen-associated molecular patterns (PAMPs) through multiple pattern recognition receptors (PRRs) such as retinoic acid inducible gene-I (RIG-I)-like receptors (RLRs) (1–3). To induce the antiviral products to jointly elicit antiviral response against invading virus-derived RNA, activated RLR receptors trigger downstream signal transduction to induce the activation of interferon regulatory factor 3/7 (IRF3/7) and result in the production of type I IFNs (4–8). The magnitude and strength of the antiviral innate immune response are modulated by both stimulatory and inhibitory signals, where dysfunctional and uncontrolled responses may be detrimental, even fatal to the host. Insufficient production of IFNs may dampen efficient viral clearance, whereas excessive IFNs production may cause tissue damage and spontaneous autoimmunity. Thus, an appropriate outcome of type I IFN signaling is required to efficiently eliminate viral invasion while avoiding harmful immunopathology (9, 10).

Tumor necrosis factor (TNF)-α-induced protein 8-like 2 (TIPE2, also known as TNFAIP8L2), one of the four members of the TIPE family, was originally identified as a novel immunoregulatory molecule that negatively regulates T-cell receptor (TCR) and Toll-like receptor (TLR) signal transduction to maintain immune homeostasis (11–13). TIPE2 is preferentially expressed in immunocytes, including lymphoid and myeloid cells in mice, while human TIPE2 is widely detected in various hematopoietic and non-hematopoietic cell types (14, 15). TIPE2 deficiency in mice spontaneously develops fatal systemic inflammation and premature death, and loss of TIPE2 augments the production of pro-inflammatory cytokines upon stimulated with various inflammatory insults (16). Accelerating evidence indicates that TIPE2 inhibits the development of a variety of malignant tumors by suppressing cell proliferation (17, 18), inducing cancer cell apoptosis (19), inhibiting tumor migration (20, 21), and promoting antitumor immune responses mediated by CD8^+^ T and natural killer (NK) cells (22). TIPE2 has been recently shown to regulate the functional polarization of myeloid-derived suppressor cells (MDSCs) and maybe a potential therapeutic target for cancer immunotherapy (23). In addition, TIPE2 has been revealed to play a role in disrupting autophagy flux *via* RAC1-MTORC1 axis and impairing autophagic lysosome reformation (24). Moreover, cells and mice lacking TIPE2 have stronger anti-bacterial ability (25). TIPE2 negatively regulates Poly (I:C) (a double-stranded RNA receptor ligand)-induced anti-RNA immune response by targeting the PI3K-Rac pathway (26). However, the physiological role and the underlying mechanism of TIPE2 in antiviral innate immunity are still obscure; rarely reports can be found in this field so far, indicating that better understanding is essential and indispensable.

In the present study, we found that vesicular stomatitis virus (VSV) infection downregulated the expression of TIPE2 in macrophages, which implied that TIPE2 may play a role in antiviral innate immunity. Significant downregulation of TIPE2 mRNA expression was detected in the peripheral blood mononuclear cells (PBMCs) from 154 cases of respiratory syncytial virus (RSV)-infected children compared with PBMCs from control healthy children. Lyz2^+^ TIPE2^f/f^ mice showed less sensitivity to VSV infection and produced more IFN-β. TIPE2 participated and interrupted TBK1-IRF3-IFN signaling pathway by directly interacting with RIG-I. Taken together, our data demonstrated that TIPE2 plays a crucial braking role in maintaining the homeostasis of the anti-RNA virus immune response by targeting RIG-I, which is conducive to the proper regulation of the innate immune defense of the host.

## Materials and Methods

### Mice and Reagents

Tumor necrosis factor-α-induced protein 8-like 2^f/f^ mice on a C57BL/6J background were generated through the CRISPR-Cas9 system to delete *Tnfaip8l2* exon 2 by Beijing Biocytogen Co. Ltd. (sgRNA sequence, sgRNA1: GCTTGTCACCCATATGAAGTTGG; sgRNA2: ACCAATGCTTCTCGATCCCCTGG). Lyz2-Cre mice on a C57BL/6J background were kindly provided by Prof. Ximei Wu, Zhejiang University School of Medicine. All mice were housed in the University Laboratory Animal Center in an environment free of specific pathogens. All animal experiments were conducted in accordance with the protocol approved by the Animal Ethics Committee from Zhejiang University School of Medicine and were in compliance with institutional guidelines.

Antibodies specific to the Myc tag (sc-40; sc-789), Flag tag (sc-807), and GAPDH (sc-130619) were from Santa Cruz Biotechnology (Delaware Ave Santa Cruz, CA, United States). Antibodies specific for TBK1 (D1B4; 3504), TBK1 phosphorylated at Ser 172 (5483), IRF3 (4962), IRF3 phosphorylated at Ser396 (4D4G; 4947), p-JNK (9251), JNK (9252), p-p65 (3033), p65 (8242), p-p38 (9215), p38 (9212), p-ERK (4370), and ERK (4695) were from Cell Signaling Technology (Danvers, MA, United States). Antibodies specific to RIG-I were from Cell Signaling Technology (D14G6), Proteintech (20566-1-AP, Proteintech Group, Inc Rosement, IL 60018, USA), and ABclonal (A0550, Wuhan, China). Antibody specific to MAVS was from Cell Signaling Technology (4983S). Antibody specific to TIPE2 was from Proteintech (15940-1-AP). TIPE2 small interfering RNA (siRNA) and control siRNA were from GenePharma (Shanghai, China). Flag-M2 Magnetic Beads (M8823) were from Sigma-Aldrich (St. Louis, MO, United States).

### Primary Cell Culture

Four days after intraperitoneal injection of thioglycollate (BD, Sparks, MD), the mouse peritoneal macrophages were collected by peritoneal lavage from mice and cultured in RPMI 1640 medium with 10% (vol/vol) fetal bovine serum (FBS) and 1% penicillin–streptomycin (P–S). Bone marrow derived macrophages (BMDMs) were generated from the bone marrow in the femurs and tibiae of 6–8 weeks old mice and differentiated in RPMI-1640 medium with 10% FBS and 20 ng/ml recombinant mouse macrophage colony-stimulating factor (R&D, 416-ML) (27, 28). Peritoneal macrophages and BMDMs were seeded in 12-well-plates at a density of 1 × 10^6^ cells/well.

### Cell Lines

RAW264.7 macrophages, HEK293T, and HeLa cells were obtained from American Type Culture Collection (ATCC) and cultured in DMEM containing 10% FBS under 5% CO2 at 37°C in a humidified incubator. THP-1 cell line was obtained from ATCC and maintained in RPMI-1640 medium with 10% FBS.

### Plasmid Constructs and Transfection

To construct recombinant vector encoding mouse TIPE2, the full-length coding region was created by PCR-based amplification of RAW264.7 complementary DNA and subcloned into the pcDNA3.1 eukaryotic expression vector (Invitrogen; Carlsbad, CA, United States). Primers for plasmid construction were: 5′-AGCTCGAGATGGAGACGTTCAGCTCCAAGGAC-3′ (forward) and 5′-TTGGTACCGATAATGTCCCGTTCTCCAGCAGTTTG-3′ (reverse). All constructs were confirmed by DNA sequencing. The Flag- RIG-I was provided by Dr Jianli Wang, Institute of Immunology, Zhejiang University. Flag-MAVS, Flag-TBK1, Flag-2CARDs, Flag-Heli, and Flag-CTD plasmids were kindly provided by Dr Xiaojian Wang, Institute of Immunology, Zhejiang University. The plasmids were transfected into cells with JetPRIME transfection reagents (Polyplus-transfection, Illkirch, France) according to the manufacturer's instructions. Primary macrophages, RAW264.7 macrophages, and THP-1 cell line were transfected with siRNA using INTERFERin reagent (Polyplus) according to the standard protocol. The sequences for TIPE2-specific siRNA are listed in Supplementary Table 1. siRNA duplexes were transfected into cells at a final concentration of 30 nM.

### Viral Infection

Respiratory syncytial virus (subtype A, strain Long) was kindly provided by Dr. Jing Qian, Zhejiang University School of Medicine. VSV-GFP was kindly provided by Dr. Zongping Xia, Life Science Institute, Zhejiang University. VSV, Sendai virus (SeV), and Herpes simplex virus type 1 (HSV-1) were gifts kindly provided by Prof. Xiaojian Wang, Zhejiang University School of Medicine. Primary macrophages were infected with RSV [multiplicity of infection (MOI) = 1] or VSV (MOI = 1) or VSV-GFP for the indicated time, and infected with VSV of indicated MOI for 9 h. BMDMs were infected with VSV (MOI = 1) for the indicated hours. THP-1 cells were infected with VSV (MOI = 1) for indicated times or VSV of indicated MOI for 9 h. Raw264.7 cells were infected with VSV (MOI 0.1) or VSV-GFP (MOI = 0.01, 0.1) for the indicated times, and infected with VSV of indicated MOI for 9 h. 293T cells were infected with VSV (MOI 0.1) or VSV-GFP (MOI = 0.01, 0.1) for indicated times, and infected with VSV of indicated MOI for 12 h. For *in vivo* studies, mice were intraperitoneally infected with VSV (10^8^ pfu per mouse) and killed 24 h after infection. For mice survival assays, 7 weeks old mice were infected with VSV (10^8^ pfu/g) *via* intraperitoneal injection.

### Quantitative Reverse Transcriptase PCR

Total RNA was extracted from cells using TRIzol reagent (Takara; Kyoto, Japan) according to the directions of the manufacturer. Single-strand cDNA was generated from total RNA using reverse transcriptase (Toyobo; Osaka, Japan). Real-time quantitative PCR analysis, using SYBR Green Master Rox (Roche), was performed as we previously described. The sequences for primers are shown in Supplementary Table 2. Data were normalized by the level of β-actin expression in each sample.

### Lung Histology

Lungs from control or virus-infected mice were dissected, fixed in 10% phosphate-buffered formalin, embedded into paraffin, sectioned, stained with hematoxylin and eosin solution, and examined by light microscopy for histological changes.

### Cytokine Release Assay

The peripheral blood of mice was collected 12 h after infection with VSV. IL-6 and IFN-β levels were detected with ELISA kits according to the protocols of the manufacturer. IL-6 ELISA kit was purchased from Invitrogen (Thermo Fisher Scientific) and IFN-β ELISA kit was purchased from InvivoGen (San Diego, CA, United States).

### Flow Cytometry

Primary macrophages were infected with GFP-VSV for the indicated time and analyzed by flow cytometry (FACS). Raw264.7 cells or HEK293T cells were seeded and incubated overnight, and then transfected as described above. After 48 h, the cells were infected with GFP-VSV for indicated time periods and subjected to flow cytometric analysis. The mean fluorescence intensity and positive percentage rate of green-fluorescent cells were determined.

### Immunofluorescence Confocal Microscopy

HeLa cells plated on glass coverslips in six-well-plates were infected or uninfected with VSV at the indicated time. Cells were fixed with 4% paraformaldehyde for 30 min, permeabilized using 0.1% Triton X-100, blocked with 1% bovine serum albumin (BSA) in phosphate-buffered saline (PBS) for 1 h, and stained with primary antibodies of rabbit anti-Myc and mouse anti-Flag, followed by stained fluorescent-dye-conjugated secondary antibodies. The nuclei were stained with DAPI (4, 6-diamidino-2- phenylindole; Sigma-Aldrich). The colocalization of Flag-RIG-I and Myc-TIPE2 was finally detected using the ZEISS LSM 880 with a fast Airy Scan microscope under a × 63 oil objective.

### Immunoprecipitation and Immunoblot Analysis

For immunoprecipitation, whole-cell extracts were lysed using IP Lysis Buffer (Pierce, 87785) supplemented with a protease inhibitor “cocktail” (Sigma, P8340). Cell lysates were centrifuged for 15 min at 12,000 g under 4°C, supernatants were collected and incubated with protein A/G magnetic beads (MedChemExpress, MCE, HY-K0202) together with specific antibodies. After overnight incubation, protein A/G magnetic beads were washed three times with IP wash buffer. Immunoprecipitates were eluted by SDS–PAGE loading buffer or Elution buffer (Pierce, 88848). For immunoblot analysis, cells were washed two times with cold PBS and lysed with cell lysis buffer (Cell Signaling Technology, 9803) supplemented with a protease inhibitor “cocktail” (Sigma, P8340). Protein concentrations of the extracts were measured by BCA assay (Pierce, 23235). Equal amounts of the extracts were loaded and subjected to SDS-PAGE, transferred onto polyvinylidene fluoride membrane (Millipore, IPVH00010), and then blotted as described previously (28). The specific protein bands were visualized by using a Pierce chemiluminescence ECL kit (Thermo Fisher Scientific, Waltham, MA, United States).

### Human Subjects and Specimens

We collected the peripheral blood samples from 154 cases of pediatric patients infected with the respiratory syncytial virus (RSV, one type of RNA virus) who admitted to Children's hospital, Zhejiang University School of Medicine, and 66 healthy children as a control group. The diagnosis of RSV infection was based on the positive results for RSV through immunofluorescence assay. Furthermore, in order to exclude other pathogens co-infections, all children were performed other microbiologic tests, including protein purified derivative (PPD), blood culture, pleural effusion and nasopharyngeal aspirate/swab cultures, nasopharyngeal aspirate/swab for virus antigens detection (respiratory syncytial viruses, influenza viruses, metapneumovirus, adenovirus, and parainfluenza virus), and serology for Chlamydia pneumoniae (CP), Chlamydia trachomatis (CT), and Legionella pneumophila (LG). No other pathogens were found by these tests. Peripheral blood samples were obtained from the patients on admission. All procedures were pre-approved by the ethics committee of the Children's Hospital, Zhejiang University School of Medicine. Written informed consent was obtained from at least one guardian of each patient before enrollment. The data from patients were analyzed anonymously.

### Statistical Analysis

Statistical analysis was carried out with GraphPad Prism 8 and all data are shown as the mean ± SEM. Statistical significance between groups was determined by two-tailed Student's *t-*test. For the mouse survival study, Kaplan–Meier survival curves were generated and analyzed for statistical significance. Values of *p* < 0.05 were considered statistically significant.

## Results

### Tumor Necrosis Factor-α-Induced Protein 8-like 2 Expression Level Decreases During RNA Viral Infection

**Figure 1:**
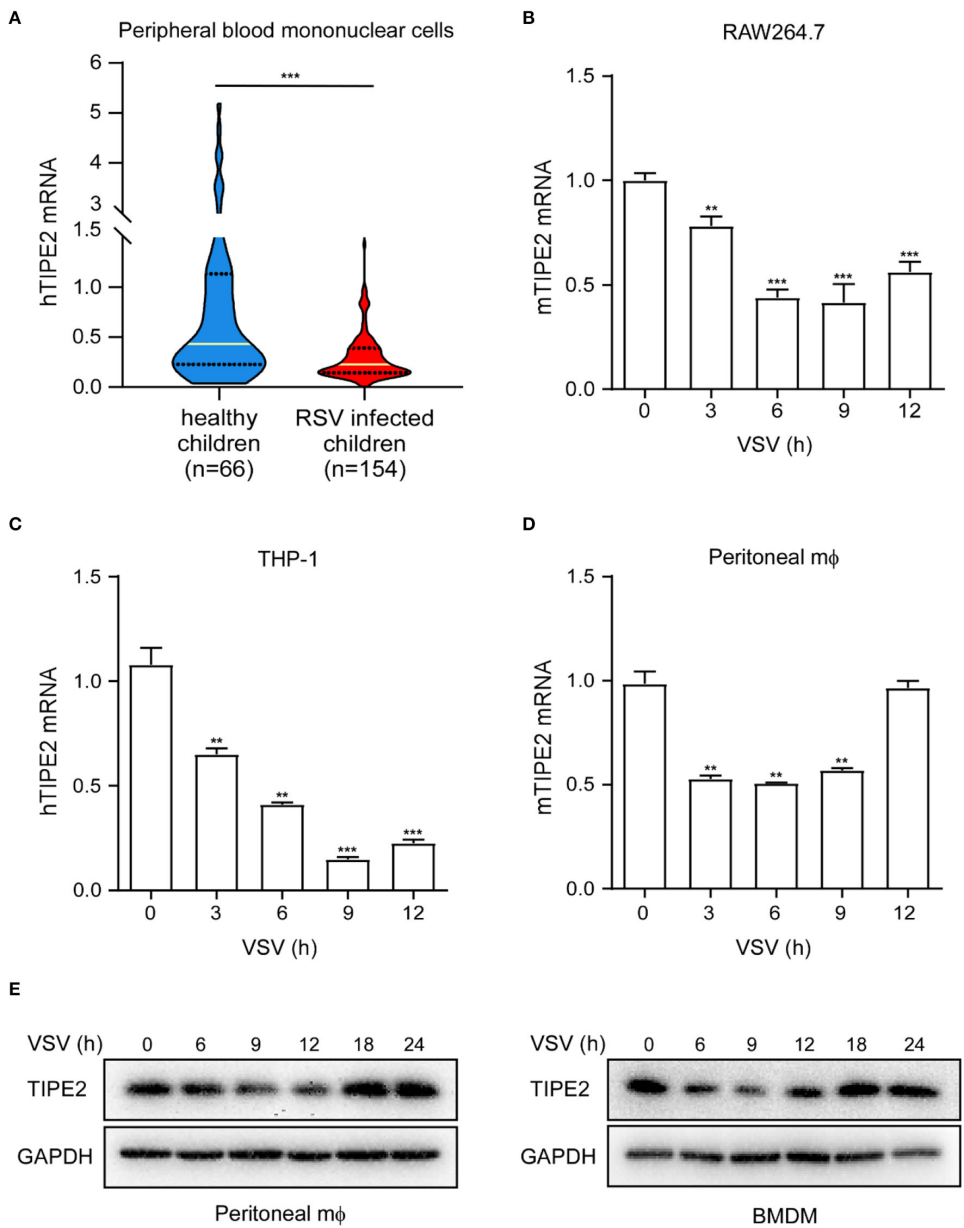
Tumor necrosis factor-α-induced protein 8-like 2 expression level decreases during RNA viral infection. **(A)** Quantitative PCR (Q-PCR) analysis of TIPE2 mRNA expression in peripheral blood mononuclear cells (PBMCs) of peripheral blood samples from 154 cases of pediatric patients infected with respiratory syncytial virus (RSV) and 66 healthy children. Q-PCR analysis of TIPE2 mRNA expression in RAW264.7 cell lines **(B)**, THP-1 cells lines **(C)**, and primary peritoneal macrophages **(D)** infected with vesicular stomatitis virus (VSV) for the indicated hours. **(E)** Immunoblot analysis of TIPE2 protein level in peritoneal macrophages and bone-marrow-derived macrophage (BMDMs) infected with VSV for the indicated hours. Data are presented as the mean ± SEM. and are representative of three independent experiments. The groups at time 3, 6, 9, and 12h are compared with time 0 group respectively in **(B–D)**. Student's t-test was used for statistical calculation. *p < 0.05, **p < 0.01, and ***p < 0.001.

To investigate whether TIPE2 is possibly involved in antiviral innate immunity, we collected the peripheral blood samples from 154 RSV infection pediatric cases and 66 healthy children as the control group. We detected the TIPE2 mRNA expression in PBMCs and found that the level in patients with RSV infection was significantly lower than that in healthy controls (Figure 1A), indicating that TIPE2 in PBMCs might correlate intimately to the antiviral immune response of the host. VSV is a prototypic RNA-enveloped virus that has been extensively used in anti-RNA virus innate immunity research due to its wide host range and highly genetic tractability (29, 30). To further confirm the downregulation of TIPE2 during the antiviral immune response, we selected macrophages including primary peritoneal macrophages, RAW264.7, and THP-1 cell lines to investigate the expression and function of TIPE2 after VSV infection. The expression of TIPE2 mRNA was downregulated after VSV infection and continued to decrease within the infection time of 0–9 h, then increased after 9 h in RAW264.7, THP-1 cell line, and primary peritoneal macrophages (Figures 1B–D). Next, we detected the expression alteration of TIPE2 protein level in peritoneal macrophages and BMDMs during VSV infection. Consistent with the above trend of results at the mRNA level, the protein level of TIPE2 gradually diminished in the time range of 0–12 h post-infection, then increased since 18 h time point (Figure 1E). These results confirmed the dynamic expression changes of TIPE2 during the early stage of viral infections, indicating that TIPE2 might function as a modulator of antiviral immune response in macrophages.

### Tumor Necrosis Factor-α-Induced Protein 8-Like 2-Deficient Mice Are More Resistant to RNA Viral Infection

**Figure 2:**
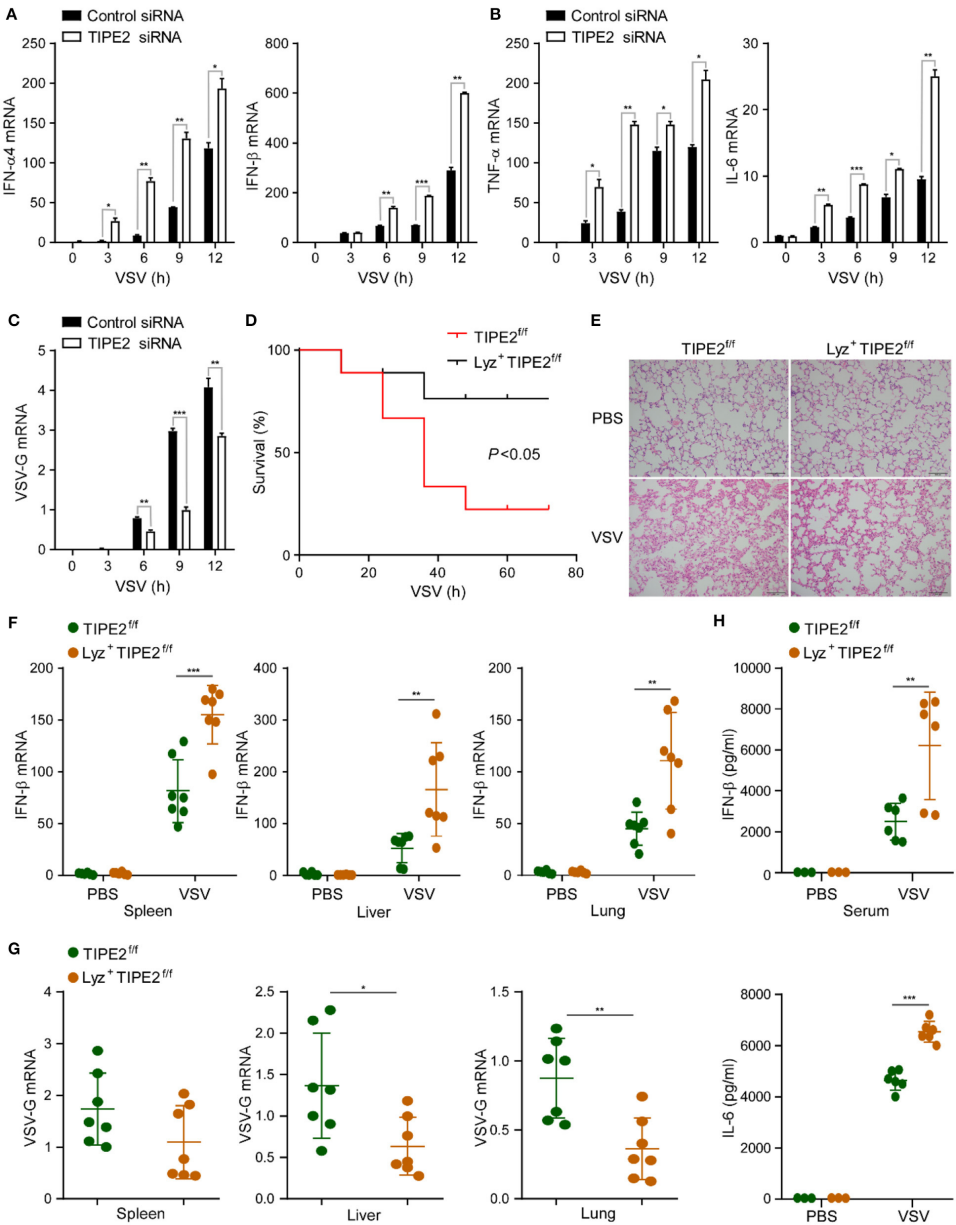
Tumor necrosis factor-α-induced protein 8-like 2-deficient mice are more resistant to RNA viral infection. **(A–C)** Q-PCR analysis of IFN-α4, IFN-β, TNF-α, IL-6, and VSV-G mRNA expression in primary peritoneal macrophages infected with VSV for the indicated hours. **(D)** Survival assay of ~8-week-old TIPE2^f/f^ and Lyz2^+^ TIPE2^f/f^ mice given intraperitoneal injection of VSV (10^8^ pfu/g) (*n* = 9 per group); *p < 0.05 (by log-rank test). **(E)** Hematoxylin and eosin staining of fractional lung tissues from TIPE2^f/f^ and Lyz2^+^ TIPE2^f/f^ mice treated with VSV (10^8^ pfu per mouse) by intraperitoneal injection. Scale bar, 100 mm. **(F)** Q-PCR analysis of IFN-β mRNA expression in the spleen, liver, and lung from mice treated as in **(E)**. **(G)** Q-PCR analysis of VSV-G expression in organs from mice treated as in **(E)**. **(H)** ELISA assay detected the production of IL-6 and IFN-β in serum from mice treated as in **(E)**. Data are presented as the mean ± SEM and are representative of three independent experiments. Student's *t*-test was used for statistical calculation. *p < 0.05, **p < 0.01, and ***p < 0.001.

To investigate the potential role of TIPE2 in virus-triggered immune response in macrophages, we silenced TIPE2 with small interfering RNA (siRNA) in peritoneal macrophages, RAW264.7, and THP-1 cell lines. After RSV infection with TIPE2 knockdown peritoneal macrophages, the expression levels of IFN-α, IFN-β, TNF-α, and IL-6 mRNA were elevated (Supplementary Figure 1A). Upon VSV challenge, TIPE2 silenced macrophages expressed higher levels of the listed cytokines and significantly decreased replication of VSV than control macrophages (Figures 2A–C, Supplementary Figures 1B–D). Based on the above data, we considered that TIPE2 negatively regulates RNA virus-triggered type I IFN and inflammatory cytokine production in macrophages.

To further confirm the immunosuppressive function of TIPE2 in antiviral innate immunity, we utilized TIPE2 conditional KO mice which were specifically targeted deletion in the myelomonocytic lineages by crossing TIPE2^f/f^ mice with Lyz2-Cre transgenic mice. Next, we investigated the physiological function of TIPE2 in host anti-RNA virus response *in vivo*. The overall survival assay suggested that knockout of TIPE2 enhanced the ability to resist VSV infection and achieved a higher survival rate compared with TIPE2^f/f^ mice (Figure 2D). Milder lung tissue damage, together with less inflammatory cell infiltration was observed in the lung tissues of Lyz2^+^ TIPE2^f/f^ mice after VSV infection (Figure 2E). Consistent with the physical condition, a marked increase of the IFN-β mRNA level was detected in the spleen, liver, and lung of Lyz2^+^ TIPE2^f/f^ mice compared to TIPE2^f/f^ mice upon VSV infection (Figure 2F). As expected, the replication of VSV was diminished in indicated organs from TIPE2 KO mice (Figure 2G). Furthermore, the levels of IFN-β and IL-6 were significantly higher in the serum of Lyz2^+^ TIPE2^f/f^ mice than TIPE2^f/f^ mice upon VSV infection (Figure 2H). These results suggested the negative regulatory function of TIPE2 in response to antiviral innate immunity *in vivo*, as individuals lacking TIPE2 demonstrating superior resistance toward VSV infection.

### Tumor Necrosis Factor-α-Induced Protein 8-Like 2 Promotes VSV Infection and Suppresses Type I IFN Production *in vitro*

**Figure 3:**
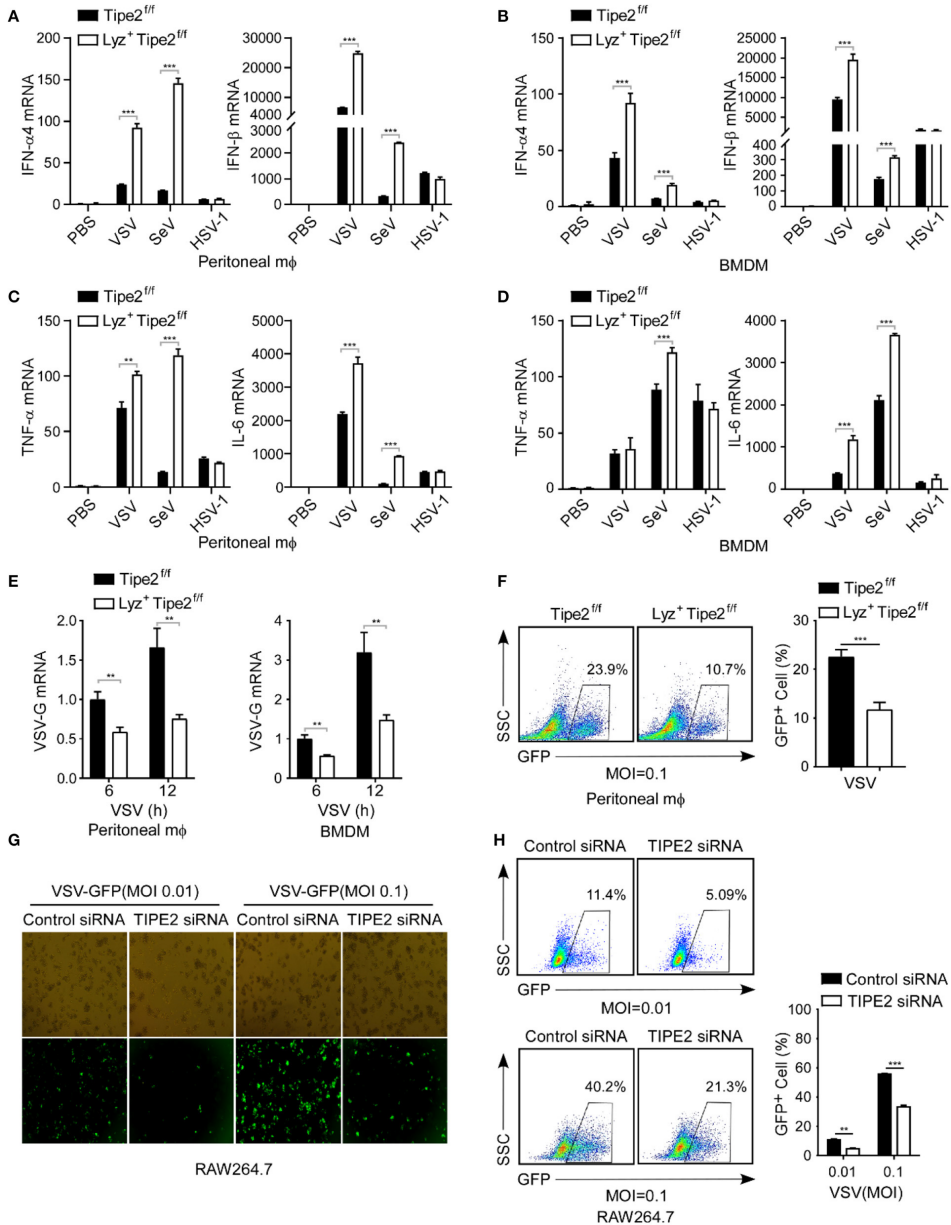
Tumor necrosis factor-α-induced protein 8-like 2 promotes VSV infection and suppresses type I IFN production *in vitro*. **(A,B)** Q-PCR analysis of IFN-α4 and IFN-β mRNA expression in TIPE2^f/f^ and Lyz2^+^ TIPE2^f/f^ peritoneal macrophages **(A)** and BMDMs **(B)** infected with VSV, SeV, or HSV-1 for 12 h. **(C,D)** Q-PCR analysis of TNF-α and IL-6 mRNA expression in TIPE2^f/f^ and Lyz2^+^ TIPE2^f/f^ peritoneal macrophages **(C)** and BMDMs **(D)** infected with VSV, SeV, or HSV-1 for 12 h. **(E)** Q-PCR analysis of VSV-G transcript in TIPE2^f/f^ and Lyz2^+^TIPE2^f/f^ peritoneal macrophages and BMDMs stimulated by VSV for 6 or 12 h. **(F)** Flow cytometry analysis of GFP fluorescence intensity and the percentage of GFP^+^ cells in peritoneal macrophages challenged with VSV-GFP (MOI = 0.1) for 12 h. **(G)** Immunofluorescence assay of VSV-GFP in RAW264.7 cells transfected with negative control mimics (nc) or TIPE2 siRNA followed by infection with VSV-GFP (MOI = 0.01 or 0.1) for 12 h. **(H)** Flow cytometry analysis of GFP fluorescence intensity and the percentage of GFP^+^ cells in RAW264.7 cells treated as in G. Data are presented as the mean ± SEM. and are representative of three independent experiments. Student's *t*-test was used for statistical calculation. *p < 0.05, **p < 0.01, and ***p < 0.001.

Type I IFNs induced by viral infection have extremely vital significance in antiviral innate immunity, for further validation, feasibility assessment regarding the inhibition of TIPE2 against virus-induced type I IFN response was implemented *in vitro*. Compared with the control peritoneal macrophages, the TIPE2-deficient peritoneal macrophages expressed significantly elevated IFN-α4 and IFN-β mRNA upon stimulation with VSV and SeV, yet failed to do so in HSV-1 infected group (Figure 3A). Consistently, there were similarly obvious increases of type I IFNs induced by VSV in BMDMs (Figure 3B). The production of pro-inflammatory cytokines, such as IL-6 and TNF-α, was also elevated in the antiviral immune response induced by VSV and SeV, instead of HSV-1 in TIPE2-deficient peritoneal macrophages and BMDMs (Figures 3C,D). The transcript level of VSV-G was markedly downregulated in TIPE2-deficient peritoneal macrophages as well as BMDMs after VSV infection (Figure 3E). By performing flow cytometry, we found that peritoneal macrophages lacking TIPE2 showed a decrease in GFP^+^ cells when infected with GFP-tagged VSV. The qualitative and quantitative analysis of VSV replication reflects the degree of viral infection across the population (Figure 3F). Correspondingly, TIPE2 silenced Raw264.7 cells showed decreased GFP^+^ cells when infected with VSV-GFP compared with control cells (Figures 3G,H). The data above further confirmed that TIPE2 negatively regulates type I IFN response and promotes viral infection in macrophages.

### Tumor Necrosis Factor-α-Induced Protein 8-Like 2 Overexpression Promotes Viral Replication by Downregulating Type I IFN Expression

**Figure 4:**
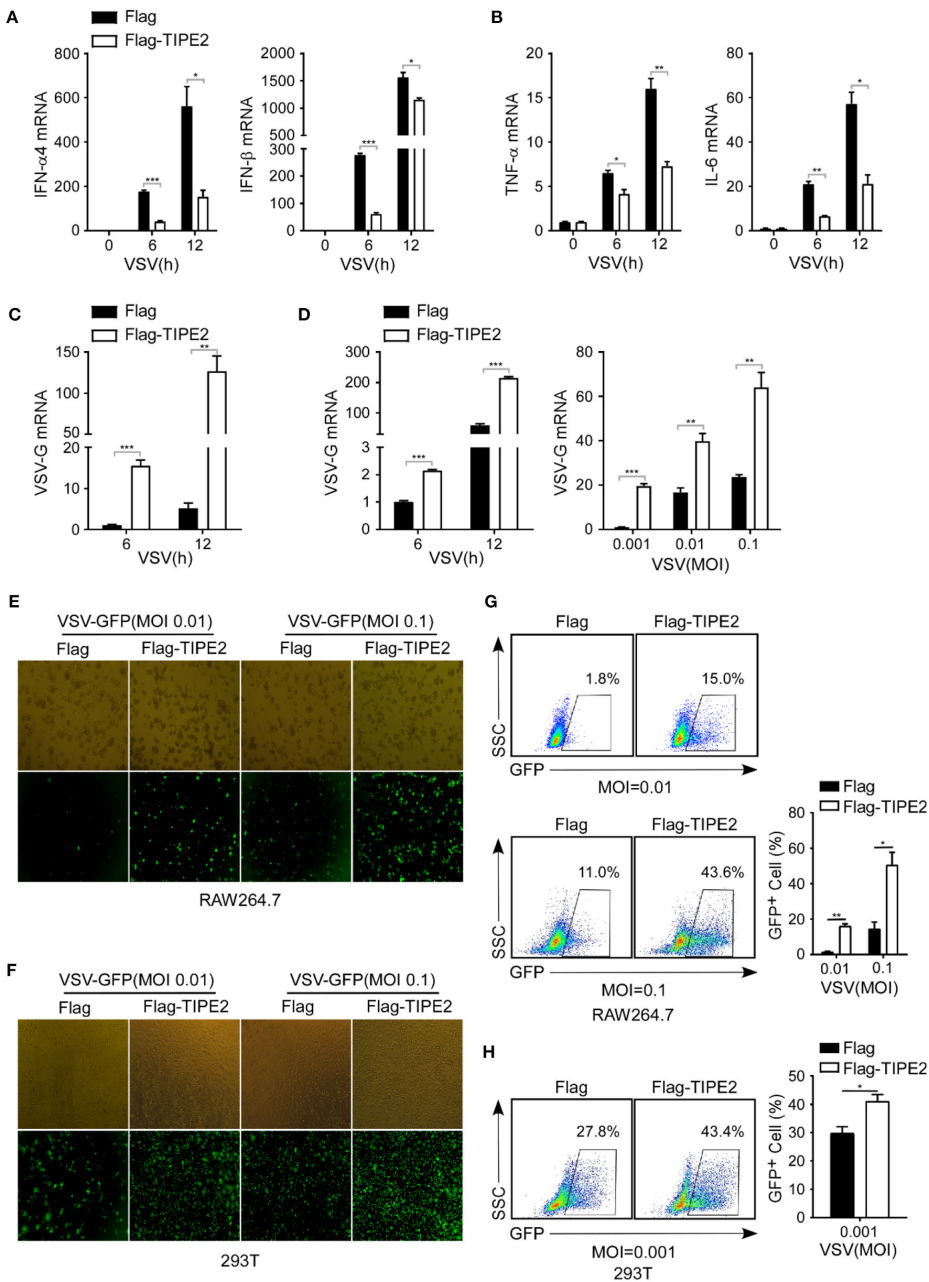
TIPE2 overexpression promotes viral replication by downregulating type I IFN expression. **(A–C)** Q-PCR analysis of IFN-α4, IFN-β, TNF-α, IL-6, and VSV-G mRNA expression in RAW264.7 cells overexpressing Flag-TIPE2 infected with VSV (MOI = 0.1) for the indicated time. **(D)** Q-PCR analysis of VSV-G transcript in HEK293T cells overexpressing TIPE2 infected with VSV (MOI = 0.01) for the indicated time or infected with VSV (MOI = 0.001, 0.01, 0.1) for 12 h. **(E,F)** Immunofluorescence assay of VSV-GFP in RAW264.7 cells **(E)** or HEK293T cells **(F)** transfected with empty vector or Flag-TIPE2, followed by infection with VSV-GFP for 12 h. **(G,H)** Flow cytometry analysis of GFP fluorescence intensity and the percentage of GFP^+^ cells in RAW264.7 cells **(G)** or HEK293T cells **(H)** transfected with empty vector or Flag-TIPE2 challenged with VSV-GFP for 12 h. Data are presented as the mean ± SEM and are representative of three independent experiments.

Next, we further examined the effect of TIPE2 on VSV replication and antiviral immune response in TIPE2 overexpressing cell lines. RAW264.7 macrophages which stably expressed TIPE2 showed significantly lower IFN-α4, IFN-β, TNF-α, and IL-6 mRNA in response to VSV infection compared to the control group (Figures 4A,B). In addition, the level of VSV-G transcript in TIPE2 overexpressing RAW264.7 and HEK293T cells was dramatically higher than that in the control group (Figures 4C,D). By applying fluorescence microscopy, the visible presence of GFP cells was more abundant in RAW264.7 cells and HEK293T cells which overexpressed Flag-tagged TIPE2 compared to control cells (Figures 4E,F). Meanwhile, flow cytometry analysis showed that the percentage of GFP-positive cells in the overexpressed group was also higher than that in the control group (Figures 4G,H). The results above elucidated that an excessive amount of TIPE2 substantially promotes the expansion of VSV virus and suppresses the type I IFN response of the host.

### Tumor Necrosis Factor-α-Induced Protein 8-Like 2 Negatively Regulates the RIG-I Signaling Pathway

Retinoic acid inducible gene-I-like receptors (RLRs) are major PRRs that mediate the antiviral response triggered by RNA viruses. The production of type I IFNs, which play a significant role in the anti-RNA virus immune response, largely depends on the signal transmission of the downstream signaling pathway of RLRs (5–7). We, therefore, investigated the potential impact of TIPE2 on important molecular events in the RLR signaling pathway.

**Figure 5:**
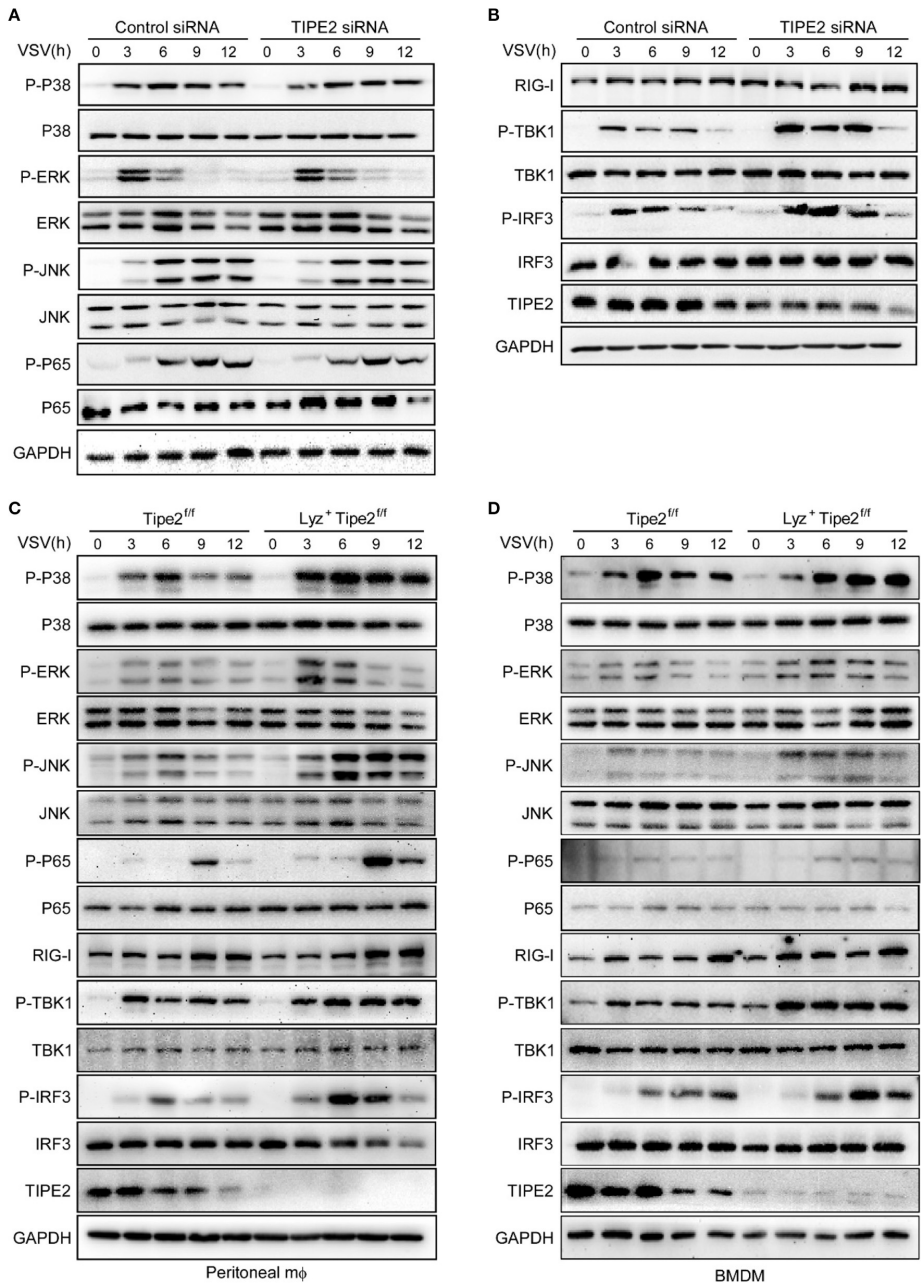
TIPE2 negatively regulates the RIG-I signaling pathway. **(A,B)** Immunoblot analysis of phosphorylated (p-) or total proteins in lysates of peritoneal macrophages transfected with nc or TIPE2 siRNA followed by infection with VSV for the indicated hours. **(C,D)** Immunoblot analysis of phosphorylated or total proteins in lysates of TIPE2^f/f^ and Lyz2^+^ TIPE2^f/f^ peritoneal macrophages **(C)** or BMDMs **(D)** infected for indicated hours with VSV.

As detected by immunoblot assay, the phosphorylation levels of TBK1 and IRF3 were significantly upregulated in TIPE2 knockdown peritoneal macrophages challenged with VSV (Figures 5A,B). Likewise, Lyz2^+^ TIPE2^f/f^ peritoneal macrophages manifested as the enhanced levels of phosphorylated TBK1 and IRF3 relative to TIPE2^f/f^ peritoneal macrophages infected with VSV (Figure 5C). Meanwhile, the phosphorylation levels of p38, ERK, and JNK were also increased in TIPE2-deficient peritoneal macrophages (Figure 5C). Similar results were also observed in Lyz2^+^ TIPE2^f/f^ BMDMs infected with VSV (Figure 5D), which together indicated an interrupted signal transduction mediated by TIPE2 upon RIG-I activation induced by RNA virus in macrophages. Taken together, our results indicated that TIPE2 possibly regulates antiviral innate immune activation by participating in the RIG-I signaling pathway and being upstream of the TBK1 signaling event.

### Tumor Necrosis Factor-α-Induced Protein 8-Like 2 Directly Interacts With RIG-I

**Figure 6:**
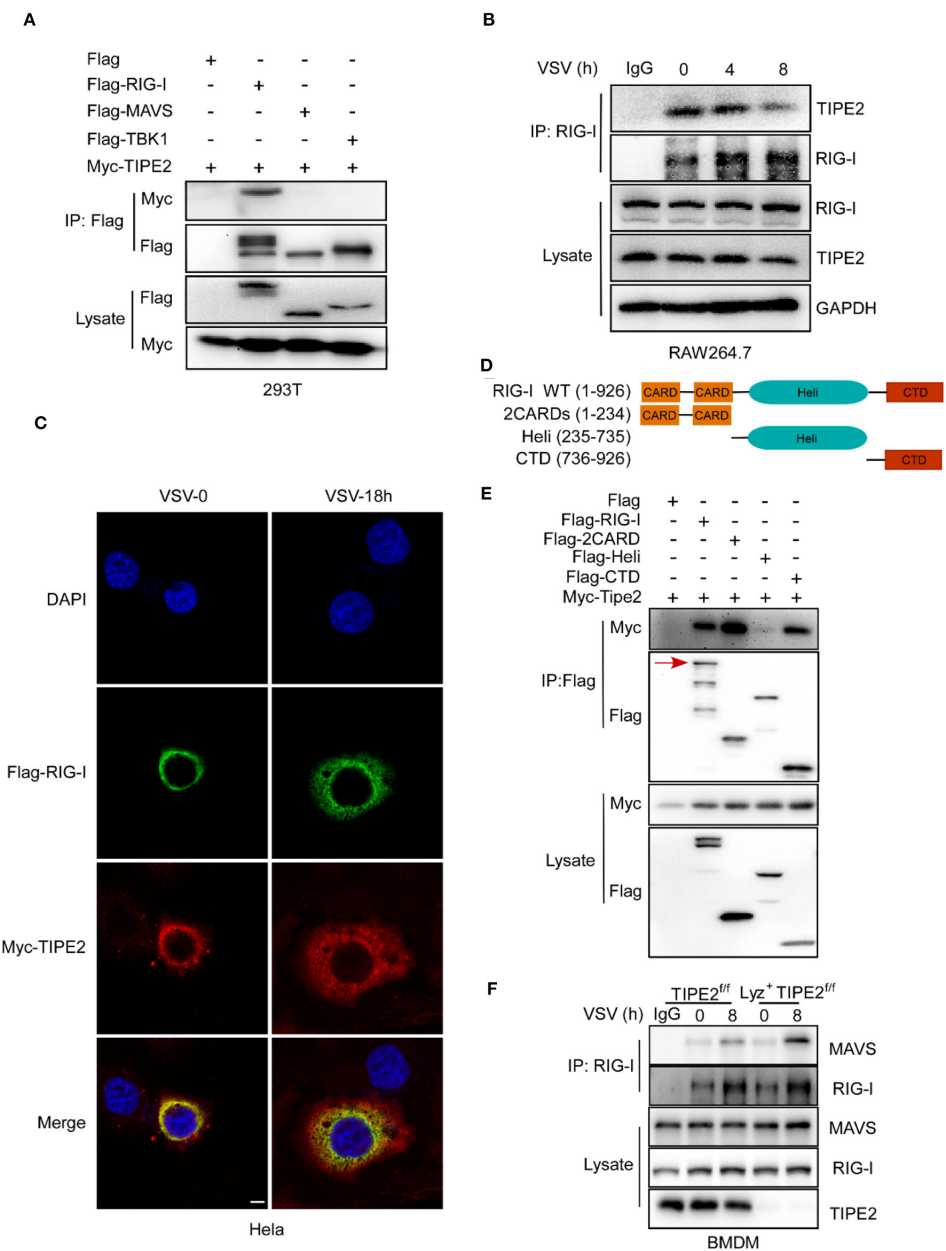
TIPE2 directly interacts with RIG-I. **(A)** Coimmunoprecipitation and immunoblot of HEK293T cells transfected with Myc-TIPE2 plasmid and empty vector (EV) or Myc-TIPE2 together with Flag-RIG-I, Flag-MAVS, or Flag-TBK1 for 24 h. **(B)** Immunoblot analysis of RAW264.7 cells infected with VSV for the indicate hours, followed by immunoprecipitation with RIG-I-conjugated agarose or immunoglobulin G (IgG)-conjugated agarose. **(C)** Confocal microscopy imaging of HeLa cells transfected with Flag-RIG-I and Myc-TIPE2 for 24 h and infected with VSV for the indicated hours. Then, the cells were labeled with antibodies against the appropriate protein and stained with DAPI for cellular nuclei. Scale bar, 5 μm. **(D)** Schematic functional structure of RIG-I and the derivatives. **(E)** Myc-TIPE2 was co-expressed with Flag-tagged full-length RIG-I (1–926), 2CARDs (1–233), Heli (234–734), or CTD (735–926) truncation structure mutants in HEK293T cells, and WCL were immunoprecipitated with anti-Flag M2 beads, followed by immunoblotting with anti-Flag or anti-Myc antibodies. **(F)** Immunoblot analysis of BMDMs from TIPE2^f/f^ and Lyz2^+^TIPE2^f/f^ mice, followed by immunoprecipitation with RIG-I-conjugated agarose or IgG-conjugated agarose. Data shown are at time 0 and 8 h. IB, immunoblot; IP, immunoprecipitation; WCL, whole cell lysate.

To further identify the effect of TIPE2 in the RLR signaling pathway, we explored whether TIPE2 interacts with several key signaling molecules. In coimmunoprecipitation (co-IP) and immunoblotting detection, three experimental groups for exogenous transfection were recruited simultaneously, with the purpose of determining the possible association between Myc-TIPE2 and Flag-RIG-I, Flag-MAVS, or Flag-TBK1. The specific band of Myc-TIPE2 was detected in the protein complex precipitated by Flag-RIG-I, which elucidated the direct binding between TIPE2 and RIG-I rather than MAVS or TBK1 (Figure 6A).

In order to further confirm the combination of TIPE2 and RIG-I, we tried to detect their endogenous binding relationship through co-IP and immunoblotting. RIG-I was enriched and immunoprecipitated from lysates of Raw264.7 cells challenged with VSV by RIG-I-conjugated agarose and immunoblot analysis was used to identify the existence of TIPE2. The result corroborated the endogenous interaction between TIPE2 and RIG-I in RAW264.7 cells (Figure 6B). Additionally, the specific binding bands of TIPE2 and RIG-I gradually faded with the prolongation of VSV infection time (Figure 6B), which is consistent with the result that TIPE2 was downregulated in response to RNA viral infection (Figure 1). The co-localization of Myc-TIPE2 and Flag-RIG-I was also observed in HeLa cells during a response to VSV infection by immunofluorescence assay, which also confirmed their connection (Figure 6C). To determine which domain of RIG-I was required for its association with TIPE2, we constructed the different deletion mutants of RIG-I, and the domain-mapping experiment showed that the caspase activation and recruitment domains (CARDs) and C-terminal domain (CTD) of RIG-I is necessary for its interaction with TIPE2 (Figures 6D,E). Collectively, the data above indicated that RIG-I interacts with TIPE2 *via* its CARDs and CTD domains.

To confirm that TIPE2 targets RIG-I to inhibit signaling molecular events downstream of the RLR pathway, we explored whether the virus-induced association between endogenous RIG-I and MAVS is enhanced in TIPE2-deficient BMDMs. The constitutive association of RIG-I-MAVS was enhanced after VSV infection, and this binding relationship was strengthened when TIPE2 was knocked out (Figure 6F). Together, these data suggested that TIPE2 functions as a negative regulator of type I IFN response by binding with RIG-I.

## Discussion

**Figure 7:**
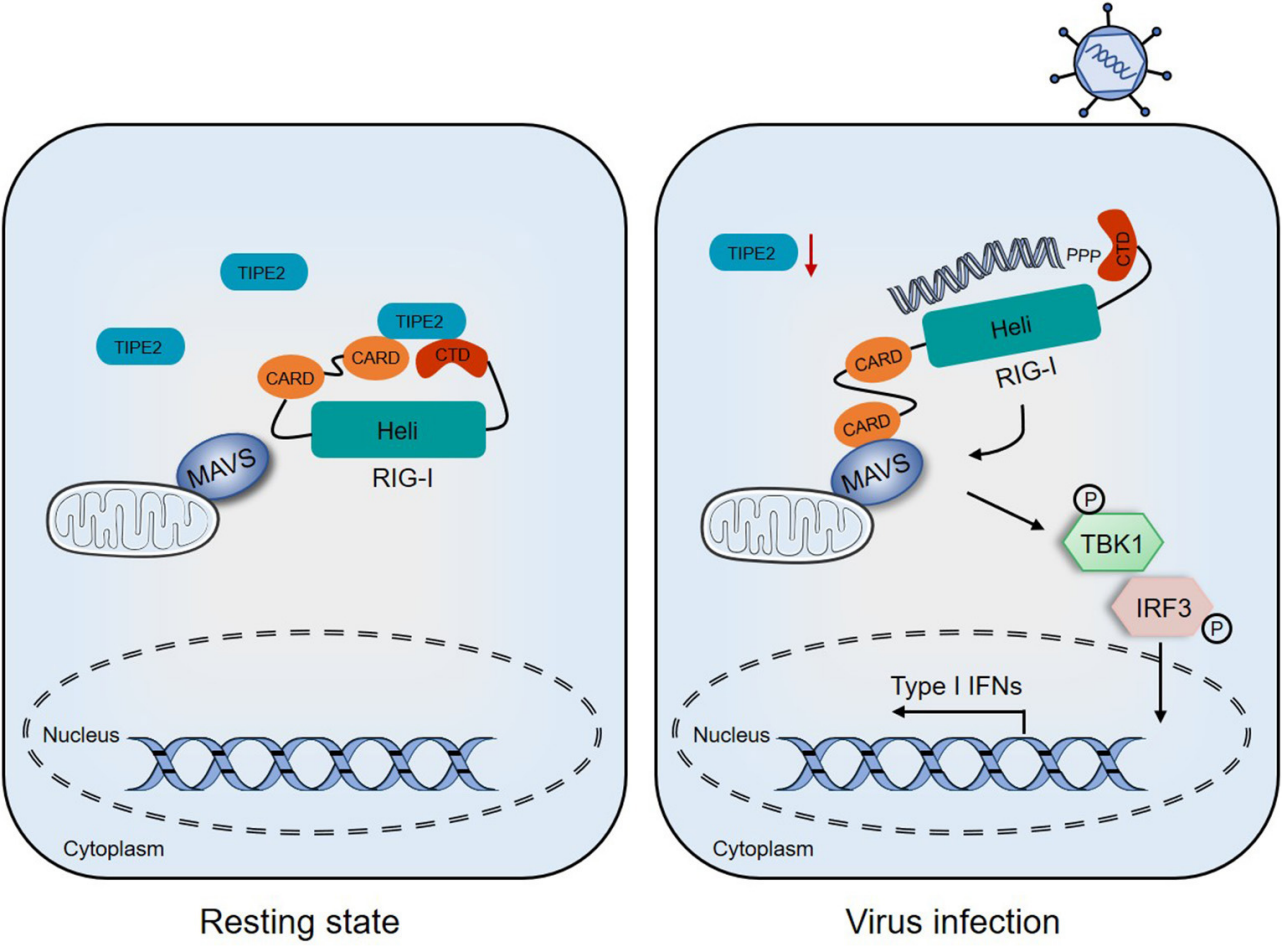
Proposed model depicting the involvement of TIPE2 in the RIG-I signaling pathway. In the resting state, TIPE2 promotes the maintenance of the inactive state of RIG-I by binding to the CARD and CTD domains. Following RNA viral infection, the decreased expression of TIPE2 may promote the conformational changes of RIG-I, which allows CARD–CARD interactions between RIG-I and MAVS. These, in turn, activate downstream signaling and ultimately establish the antiviral state of the host.

In the current study, we clarified one of the pleiotropic functions of TIPE2, where, it participates in the RIG-I-mediated anti-RNA virus innate immune response, which reveals a new regulatory signaling that serves as a significant composition of host defense against RNA viral infection (Figure 7).

Following the discovery of TIPE2 due to its elevated expression in the spinal cord tissue of mice with experimental autoimmune encephalomyelitis (EAE) (31), a growing body of evidence supports that the expression abnormality of TIPE2 plays a certain role in the pathogenesis of various inflammatory and allergic diseases and autoimmune disorders (32–34). It has been demonstrated that the TIPE2 level is reduced in the PBMCs of patients with systemic lupus erythematosus (SLE), and collagen-induced arthritis (CIA) mice with a negative correlation with the development of arthritis (35, 36). The significant increase in TIPE2 expression in patients with ankylosing spondylitis (AS) is accompanied by a negative correlation with the expression of inflammatory cytokines, which is also speculated to maintain immune homeostasis and inhibit a hyperinflammatory response (37). In chronic hepatitis infection, hepatitis B and C viruses attenuate the expression of TIPE2 and promote the occurrence of chronic hepatitis (38, 39). Stimulation of RNA such as Poly (I:C) has also been shown to provoke the paradoxically downregulation of TIPE2, which also concludes that TIPE2 functions as a negative regulatory factor in innate immunity (26). In addition, the aberrant presence of TIPE2 plays an important physiological role in the onset, development, and progression of diabetic nephropathy (40), atherosclerosis (41), stroke (42), and carcinoma (43, 44). In our present study, we determined that TIPE2 mRNA expression in PBMCs of patients with RSV infection was decreased obviously (Figure 1A). In several macrophages including primary peritoneal macrophages, RAW264.7, and THP-1 cell lines, it was further observed that TIPE2 mRNA level decreased initially and then increased during VSV infection (Figures 1B–D). At the protein expression level, the variation of TIPE2 is a similar dynamic trend (Figure 1E), which together represents that TIPE2 is also anomalous and likely performing certain functions in RNA viral infectious diseases. Based on functional verification, we speculate that the expression of TIPE2 is repressed during RNA viral infection, which is beneficial for inhibiting virus amplification by activating the RLR signaling pathway. However, there are still many doubts about the mechanism of downregulating TIPE2 expression during RNA viral infection. In our research, TIPE2 knockout macrophages in mice are more resistant to VSV stimulation, producing more type I IFNs and pro-inflammatory cytokines than the wild-type macrophages. Therefore, the expression of TIPE2 is closely associated with the production of type I IFNs and may be involved in the feedback regulation. In addition, whether the transcriptional inhibition of TIPE2 gene is related to transcription factors including IRF3 and NF-κB, and whether the downregulation of TIPE2 protein level has some connection with protein modification such as ubiquitination remain to be further studied.

Tumor necrosis factor-α-induced protein 8-like 2 is involved in the regulation of various physiological functions including inflammation, immunity, apoptosis, and cancer (11–13, 17–21). It has been reported that TIPE2 is a negative regulator required for maintaining immune homeostasis *via* inhibiting the activation of activator protein 1 (AP-1), NF-κB, and MAPK to negatively regulate the responses mediated by TCR and TLR (11, 45, 46). TIPE2 has served as a novel target for therapeutic prevention in multiple inflammatory diseases, and potential intervention to break immune tolerance may be necessary (12, 13). In innate immunity against infections, TIPE2 has been reported to reduce phagocytosis and oxidative burst and may be targeted to effectively resist bacterial infections (25). TIPE2 inhibits the expression of cytokines (including IFN-β and IL-6) mediated by Poly (I:C) in innate immune response in a PI3K-Rac pathway-dependent manner (26). However, the role of TIPE2 in anti-virus, especially RNA virus innate immune activation, has not been discussed. In our study, we have supplemented the new function of TIPE2 in anti-RNA virus innate immunity, which adds new insights to understand the immunoregulatory function of TIPE2.

Diverse physiological reactions are composed of complex functional networks, and the process of TIPE2 completing different instructions is often accompanied by cooperation with multiple partners. TIPE2 was originally proved to be a caspase-8 binding protein, which regulates caspase-8-dependent functions (11). The close connection between TIPE2 and caspase-8 promotes Fas-induced apoptosis, while inhibiting AP-1 and NF-κB signaling (11, 19). Simultaneously, the negative regulation of TCR and TLR signaling by TIPE2 also targets the caspase-8-containing complex (11). TIPE2 enhances the oxidative burst and phagocytosis strength of macrophages in innate immunity through binding with Rac GTPases and blocking Rac activation and downstream Rac-PAK signaling (25, 39). TIPE2 was also found to negatively control MTOR activity *via* binding to Rac1 competed with MTOR, thereby affecting autophagy flu and impairing autophagic lysosome reformation (24). Furthermore, the endogenous interaction between TIPE2 and TAK1 prevents the formation of the TAK1-TAB1-TAB2 signal complex, which blocks TAK1 kinase activity and TAK1-NF-κB mediated inflammatory response (47). Recent research also pointed out that TIPE2 can interact with β-catenin to suppress the migration and invasion of endometrial cells by reversing epithelial-mesenchymal transition (21). The results in our study verified RIG-I as a new binding chaperone of TIPE2 and indicated that TIPE2 participates in the antiviral innate immune response by targeting RIG-I. According to the domain-mapping experiment, we further speculated that TIPE2 promotes the maintenance of the inactive state of RIG-I by binding to the CARD and CTD domain.

In summary, we propose the following working model of TIPE2, that the reduction of TIPE2 in the early stage of the response boosts the activation of RLR signaling to limit RNA viral infections. During the subsequent recovery of the response, surged TIPE2 targets RIG-I to interfere with downstream cascade signaling, which ultimately moderates the immune response. The novel mechanism discovery of TIPE2 in antiviral immunity provides new insights to avoid the toxic side effects of IFNs and crippling autoimmune diseases.

## Data Availability Statement

The original contributions presented in the study are included in the article/Supplementary Material, further inquiries can be directed to the corresponding author/s.

## Ethics Statement

The studies involving human participants were reviewed and approved by the ethics committee of the Children's Hospital, Zhejiang University School of Medicine. Written informed consent to participate in this study was provided by the participants' legal guardian/next of kin. The animal study was reviewed and approved by the Animal Ethics Committee from Zhejiang University School of Medicine.

## Author Contributions

YZha and YS designed and supervised the research. ZZ wrote the manuscript. ZZ, ML, YZho, JL, and TP performed the experiments and data analysis. QiW, LL, and YS helped with manuscript editing. LZ and QuW helped with infections of mice and related analysis. All authors reviewed and approved the manuscript.

## Conflict of Interest

The authors declare that the research was conducted in the absence of any commercial or financial relationships that could be construed as a potential conflict of interest.
