# Supplementary material for: Tumor Necrosis Factor-α-Induced Protein 8-Like 2 Negatively Regulates Innate Immunity Against RNA Virus by Targeting RIG-I in Macrophages
Source: Front Immunol. 2021 Mar 19;12:642715. doi: 10.3389/fimmu.2021.642715 (PMC8017232; doi:10.3389/fimmu.2021.642715)
Supplement: Supplementary file 1 [file Data_Sheet_1.pdf]

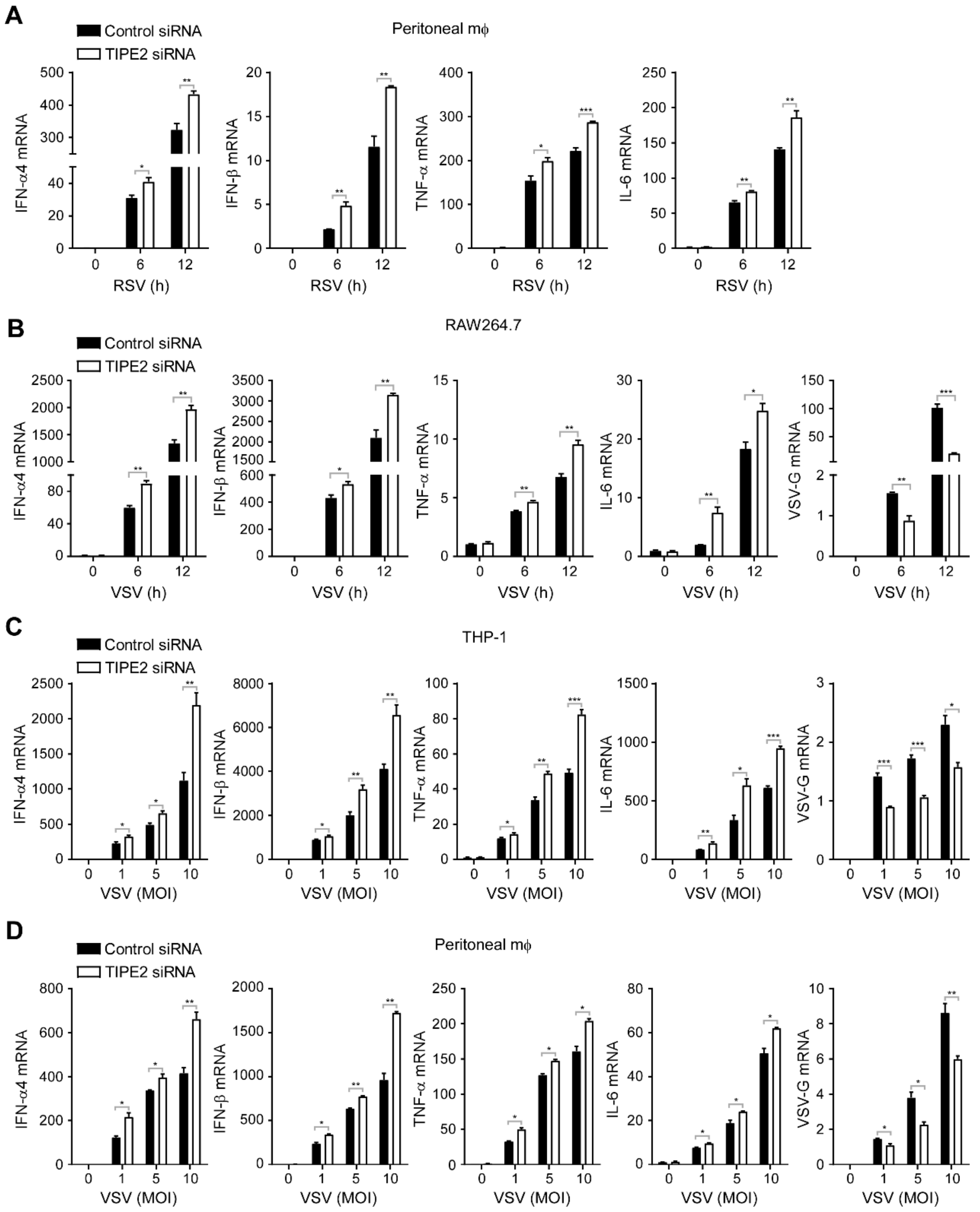

Supplementary Figure 1. Macrophages lacking TIPE2 are more resistant to RNA virus infection

(A) Q-PCR analysis of IFN-α4, IFN-β, TNF-α, and IL-6 mRNA expression in peritoneal macrophages infected with RSV for the indicated hours. (B-D) Q-PCR analysis of IFN-α4, IFN-β, TNF-α, IL-6 and VSV-G mRNA expression in RAW264.7 cells (B), THP-1 cells (C) and primary peritoneal macrophages (D) infected with VSV at a specific MOI values (MOI=1, 5, 10) for 12h or infected with VSV for the indicated

hours. Data are presented as the mean ± s.e.m. and are representative of three independent experiments. Student's *t* test was used for statistical calculation. $*P < 0.05$, $**P < 0.01$ and $***P < 0.001$.

Supplementary Table 1. The sequences for mouse TIPE2-specific siRNA.

siRNA-166	sense	CCGUGGCGCAUCUCUUUAUTT
	antisense	AUAAAGAGAUGCGCCACGGTT
siRNA-351	sense	GCUACACGAUUUCGUCAGATT
	antisense	UCUGACGAAAUCGUGUAGCTT
siRNA-517	sense	GCAUCAGGCACGUGUUUGATT
	antisense	UCAAACACGUGCCUGAUGCTT
control siRNA	sense	UUCUCCGAACGUGUCACGUTT
	antisense	ACGUGACACGUUCGGAGAATT

Supplementary Table 2. Primers for RT-PCR.

h β-actin	F(forward)	CATGTACGTTGCTATCCAGGC
	R(reverse)	CTCCTTAATGTCACGCACGAT
m β-actin	F	AGTGTGACGTTGACATCCGT
	R	GCAGCTCAGTAACAGTCCGC
h TIPE2	F	GGAACATCCAAGGCAAGACTG
	R	AGCACCTCACTGCTTGTCTCATC
m TIPE2	F	TCTCAGAAACATCCAAGGCC
	R	TTTGAGCTGAAGGACTCCATG
m IFN-α4	F	TACTCAGCAGACCTTGAACCT
	R	CAGTCTTGGCAGCAAGTTGAC
m IFN-β	F	ATGAGTGGTGGTTGCAGGC
	R	TGACCTTTCAAATGCAGTAGATTCA
m TNF-α	F	TTAAAAACCTGGATCGGAACCAA
	R	GCATTAGCTTCAGATTTACGGGT

m IL-6	F	TAGTCCTTCCTACCCCAATTTCC
	R	TTGGTCCTTAGCCACTCCTTC
VSV-G	F	ACGGCGTACTTCCAGATGG
	R	CTCGGTTCAAGATCCAGGT
